# Supplementary material for: Modulation of antibiotic effects on microbial communities by resource competition
Source: Nat Commun. 2023 Apr 26;14:2398. doi: 10.1038/s41467-023-37895-x (PMC10133249; doi:10.1038/s41467-023-37895-x)
Supplement: Supplementary file 1 — Supplementary Information [file 41467_2023_37895_MOESM1_ESM.pdf]

Supplementary Information for "Modulation of antibiotic effects on microbial communities by resource competition"

Daniel Philip Newton[1,2], Po-Yi Ho[1,*], Kerwyn Casey Huang[1,3,4,*]

[1]Department of Bioengineering, Stanford University, Stanford, CA, USA
[2]Department of Physics, Stanford University, Stanford, CA, USA
[3]Department of Microbiology and Immunology, Stanford University School of Medicine, Stanford, CA 94305, USA
[4]Chan Zuckerberg Biohub, San Francisco, CA 94158

[*]Correspondence to: poyiho@stanford.edu and kchuang@stanford.edu

This PDF contains:

- Supplementary Text
- Supplementary Figures S1-S6

SUPPLEMENTARY TEXT

Model details

We build on the consumer-resource (CR) model in Ref. 29, which presents a model for m species competing for p types of steadily supplied resources at rates $\vec{s} = (s_1, \ldots, s_p)$ where $\sum_{\mu=1}^{p} s_\mu = S$ and s_μ is the supply rate of resource μ. The dilution rate of the chemostat is d but the resource loss rates (due to efflux from the chemostat) are approximated to be zero; this approximation simplifies calculations and has been shown to not significantly affect population dynamics. Species i has resource consumption rates $\vec{R}_i = (R_{i1}, \ldots, R_{ip})$, and enzyme budget $E_i = \sum_{\mu=1}^{p} R_{i\mu}$, where $R_{i\mu}$ is the rate at which species i consumes resource μ. As stated in the main text, we begin with a classic formulation of a well-mixed CR model in a chemostat with dilution rate d,

$$\frac{dn_i}{dt} = (g_i(\vec{n}) - d)n_i, \quad (1)$$

where n_i is the abundance of species i, $\vec{n} = (n_1, \ldots, n_m)$ is the vector of species abundances, and g_i is the growth rate of species i, given by

$$g_i(\vec{n}) = \sum_{\mu=1}^{p} R_{i\mu} c_\mu, \quad (2)$$

where c_μ is the concentration of resource μ:

$$c_\mu = \frac{s_\mu}{\sum_{k=1}^{m} n_k R_{k\mu}}. \qquad (3)$$

Combining Eq. 1-3 results in a single equation governing the population dynamics when all species have uniform 'death' rate d (due to dilution):

$$\frac{dn_i}{dt} = n_i \left(\sum_{\mu=1}^{p} \frac{R_{i\mu} s_\mu}{\sum_{k=1}^{m} n_k R_{k\mu}} - d \right). \qquad (4)$$

Introduction of species-specific death rates d_i to model perturbation by bactericidal antibiotics

To model bacteriostatic antibiotics, we assumed that the consumption rates $R_{i\mu}$ of species i decrease by a factor b_i, leading to the following modification of Eq. 4:

$$\frac{dn_i}{dt} = n_i \left(\sum_{\mu=1}^{p} \frac{(R_{i\mu}/b_i) s_\mu}{\sum_{k=1}^{m} n_k (R_{k\mu}/b_k)} - d \right). \quad (5)$$

To model the effects of bactericidal antibiotics, we assumed that species i experiences death at rate d_i in addition to the effects of dilution, leading to the following modification of Eq. 4 to include both the dilution rate and the species-specific death rate d_i:

$$\frac{dn_i}{dt} = n_i\left(\sum_{\mu=1}^{p} \frac{R_{i\mu}s_\mu}{\sum_{k=1}^{m} n_k R_{k\mu}} - (d + d_i)\right). \qquad (6)$$

Note that throughout the text, we refer to the death rate as the sum of the dilution rate and the species-specific death rate. The two seemingly different modifications to the CR model in Eq. 5 and 6 can be related by a transformation of the consumption rates $R'_{i\mu} = R_{i\mu}/b_i$, where $b_i = 1 + d_i/d$, as well as a change of variables $n_i \equiv n'_i/b_i$. Plugging this change of variables into Eq. 6,

$$\frac{d(n'_i/b_i)}{dt} = \frac{n'_i}{b_i}\left(\sum_{\mu=1}^{p} \frac{R_{i\mu}s_\mu}{\sum_{k=1}^{m} n'_k R_{k\mu}/b_k} - (d + d_i)\right),$$

from which it follows that,

$$\frac{dn'_i}{d\tau_i} = n'_i\left(\sum_{\mu=1}^{p} \frac{R'_{i\mu}s_\mu}{\sum_{k=1}^{m} n'_k R'_{k\mu}} - d\right) \qquad (7)$$

for time rescaling $\tau_i \equiv tb_i$ and scaling of the resource consumption rates $R'_{i\mu} \equiv R_{i\mu}/b_i$. The solutions to Eq. 5 and Eq. 7 are identical, up to a scaling of the amplitudes ($n'_i \equiv n_i b_i$) and a scaling of time ($\tau \equiv tb_i$). Therefore, since $b_i \neq 0$, a set of species will coexist in the scenario governed by Eq. 5 if and only if that set of species coexists in the scenario governed by Eq. 7.

To summarize, consider a set $\mathcal{C}$ of p supplied resources and m species with resource consumption rates $R_{i\mu}$ and species-specific death rates $d_i + d$. Consider the transformation $\mathcal{C} \mapsto \mathcal{C}'$ defined by a transformation of the consumption rates and death rates,

$$R_{i\mu} \mapsto R'_{i\mu} = \frac{dR_{i\mu}}{d+d_i} \text{ and } d_i + d \mapsto d. \, (8)$$

This transformation preserves the property of coexistence, i.e., species $i \in \mathcal{C}$ coexists ($n_i^* > 0$) if and only if species $i \in \mathcal{C}'$ coexists. In other words, for a community with uniform death rates d in steady state that is perturbed by bactericidal antibiotics (i.e., the death rate of species i becomes $d + d_i$), we can view this perturbation as simply a transformation of the species i enzyme budget of $E_i \mapsto E'_i = dE_i/(d + d_i)$ when

considering the effect of the perturbation on coexistence. We therefore consider communities with uniform death rate d when proving the coexistence rules because these rules can apply to communities with species-specific death rates using the transformation given by Eq. 8.

Proof of coexistence rules for m species, p resources, arbitrary enzyme budgets, and uniform death rates d

Lemma 1: *If the consumption rates of two species A and B are linearly dependent, i.e., for all resources μ, $R_{A\mu} = cR_{B\mu}$ for some positive constant $c \neq 1$, then species A and B cannot coexist if they have equal death rates d.*

Proof: For species A and B to coexist, their steady state growth rates g_A and g_B, respectively, must be equal to their death rates $d_A = d$ and $d_B = d$, respectively. However, this equality cannot occur since g_A cannot equal g_B due to the relation $g_A = \sum_{\mu=1}^{p} R_{A\mu}\, c_\mu = \sum_{\mu=1}^{p} cR_{B\mu}\, c_\mu = cg_B \neq g_B$. Therefore, in what follows, we assume no pair of species strategies are linearly dependent.

Lemma 2: *Species A and B with equal death rate d cannot coexist if $R_{A\mu} > R_{B\mu}$ for all resources μ.*

Proof: Consider the growth rate of species A and B to be g_A and g_B, respectively. For the two species to coexist, g_A and g_B must equal the death rate d at steady state. However, g_A cannot equal g_B, because $g_A = \sum_{\mu=1}^{p} R_{A\mu}\, c_\mu > \sum_{\mu=1}^{p} R_{B\mu}\, c_\mu = g_B$ since the resource concentrations c_μ must be positive. Therefore, in what follows, we assume no pair of species A and B satisfies $R_{A\mu} > R_{B\mu}$ for all resources μ.

Lemma 3: *The resource consumption rates $R_{i\mu}$ of a set of coexisting species with equal death rates d must obey a linear metabolic tradeoff whereby there exist steady state nutrient concentrations $c_\mu^* > 0$ such that $\sum_{\mu=1}^{p} R_{i\mu}\, c_\mu^* = d$ for all species i.*

Proof: Since the supply rate of all resources is positive by assumption, by Eq. 3, the nutrient concentrations c_μ must always be positive, from which it follows that the steady-state nutrient concentrations c_μ^* must be positive. Furthermore, by Eq. 1, all species coexist when the steady state growth rates g_i^* satisfy $g_i^* = d$. Thus, by Eq. 2, there must exist steady-state resource concentrations $c_\mu^* > 0$ such that $g_i^* = \sum_{\mu=1}^{p} R_{i\mu} c_\mu^* = d$ if the set of species coexist.

We can interpret this coexistence condition as a geometric constraint on the resource consumption strategy vectors $\vec{R}_i$ in the space of resource consumption rates as follows. If all species coexist, then since $\sum_{\mu=1}^{p} R_{i\mu} c_\mu^* = d$, all resource consumption strategies $\vec{R}_i$ lie on the p-dimensional hyperplane in the space of resource consumption rates defined by its axis intercepts, where the intercept of the axis corresponding to resource μ is d/c_μ^*.

Lemma 4: *For p resources and m species with equal death rates d, all species coexist if and only if there exist $c_\mu^* > 0$ such that $\sum_{\mu=1}^{p} R_{i\mu}\, c_\mu^* = d$ for all species i and the normalized supply rate vector $\hat{s} \equiv (d/S)\vec{s}$ lies in the convex hull of the rescaled consumption strategies $\vec{\tilde{R}}_i = \left(c_1^* R_{i1}, \ldots, c_p^* R_{ip}\right)$.*

Proof: There are two cases: $m \geq p$ and $m < p$, and we consider each separately. The statement was proved for the case $m \geq p$ in [1]. Here, we prove the statement for the other case, $m < p$; our proof holds for $m = p$ as well.

For coexistence of all species in the community, we need $dn_i/dt = 0$ at steady-state abundances $n_i^* > 0$, so by Eq. 1, this requirement is equivalent to $g_i(\vec{n}^*) = d$, or

$$d = \sum_{\mu=1}^{p} R_{i\mu}\, c_\mu^*, \qquad (9)$$

where

$$c_\mu^* = \frac{s_\mu}{\sum_{k=1}^{m} n_k^* R_{k\mu}}. \qquad (10)$$

For coexistence of all species, we need a solution $n_i^* > 0$ and $c_\mu^* > 0$. Since Eq. 9 must hold for all species i, it represents m equations, and since all resource consumption

strategies $\vec{R}_i$ are linearly independent, we are left with $p-m$ free variables. Rewriting c_μ^* in terms of the $p-m$ free variables and plugging into Eq. 10, we now have p unknowns since we must solve for the m values of n_i^* and the $p-m$ free variables from Eq. 9. Since Eq. 10 represents p distinct equations due to the linear independence of $\vec{R}_i$, there are two cases: there is a unique solution for the values of $n_i^* > 0$ and $c_\mu^* > 0$, or there is not. Note that we used the linear independence of $\vec{R}_i$ and the fact that we have a total of $m+p$ distinct equations and $m+p$ unknowns to rule out the possibility that there are an infinite number of solutions.

Defining the rescaled consumption rate vector $\vec{\hat{R}}_i = \left(c_1^* R_{i1}, \dots, c_p^* R_{ip}\right)$ and rearranging Eq. 10, in vector notation, we conclude that all species coexist if the system of equations

$$n_1^* \vec{\hat{R}}_1 + \cdots + n_m^* \vec{\hat{R}}_m = \vec{s}, \qquad (11)$$

has a positive solution $n_i^* > 0$. It follows from Eq. 9 and 10 that $\sum_{k=1}^{m} n_k^* = S/d$, so we define the normalized supply rate vector $\hat{s} \equiv (d/S)\vec{s}$ and observe that Eq. 11 having a positive solution is equivalent to the following equation having a positive solution:

$$n_1^* \vec{\hat{R}}_1 + \cdots + n_m^* \vec{\hat{R}}_m = \hat{s}, \text{ where } \sum_{i=1}^{m} n_i^* = 1, \qquad (12)$$

which is equivalent to $\hat{s}$ lying in the convex hull of the vectors $\vec{\hat{R}}_i$. Note that the convex hull normally includes nonnegative solutions $n_i^* \geq 0$ of Eq. 12, but we assume that no values of n_i^* are zero, i.e., we assume that $\hat{s}$ does not lie on the perimeter of the convex hull of the vectors $\vec{\hat{R}}_i$.

We have shown that there exists a unique steady-state solution $n_i^* > 0$ if and only if $\hat{s}$ lies in the convex hull of the vectors $\vec{\hat{R}}_i$ for positive steady-state nutrient consumption rates satisfying $d = \sum_{\mu=1}^{p} R_{i\mu} c_\mu^*$. We next show that the unique steady state solution n_i^* is an attractor of the system.

We write $\vec{n} = \vec{n}^* + \Delta\vec{n}$ and linearize the growth equations in Eq. 1-3 around the unique fixed point $\vec{n}^*$:

$$\frac{d\Delta n_i}{dt} \approx \left(\sum_{\tau=1}^{m} \frac{\partial g_i}{\partial n_\tau}(\vec{n}^*)\Delta n_\tau\right) n_i^*. \qquad (13)$$

Assuming coexistence, and therefore a unique steady state solution n_i^*, c_μ^*, we can treat these as known constants that do not depend on $\Delta\vec{n}$. We next calculate the partial derivatives of the growth rate at $\vec{n}^*$,

$$\frac{\partial g_i}{\partial n_\tau}(\vec{n}^*) = -\sum_{\mu=1}^{p} R_{i\mu} R_{\tau\mu} \frac{s_\mu}{\left(\sum_{k=1}^{m} n_k^* R_{k\mu}\right)^2},$$

then plug into Eq. 13 to obtain the linearized system of growth equations,

$$\left(\frac{d\Delta n_1}{dt}, \dots, \frac{d\Delta n_m}{dt}\right)^T = -DM(\Delta n_1, \dots, \Delta n_m)^T,$$

where $D \equiv \text{diag}(\vec{n}^*)$, and $M_{ij} \equiv \sum_{\mu=1}^{p} R_{i\mu} R_{j\mu} \frac{s_\mu}{\left(\sum_{k=1}^{m} n_k^* R_{k\mu}\right)^2}$. We next argue that the eigenvalues of DM are nonnegative, from which it follows that the unique solution $n_i^* > 0, c_\mu^*$ is an attractor of the system.

Here, we use an argument similar to Ref. [29]. Since $n_i^* > 0$, D is invertible, so the transformation $DM \mapsto D^{1/2} M D^{1/2}$ is a similarity transformation, from which it follows that the eigenvalues of $D^{1/2} M D^{1/2}$ are identical to those of DM. For arbitrary vector $\vec{v}$,

$$\vec{v}^T D^{1/2} M D^{1/2} \vec{v} = \sum_{\mu=1}^{p} \frac{\left(\sum_{k=1}^{m} n_k^* R_{k\mu}\right)^2}{s_\mu} \left(\sum_{i=1}^{m} v_i R_{i\mu} \sqrt{n_i^*}\right) \left(\sum_{j=1}^{m} v_j R_{j\mu} \sqrt{n_j^*}\right) =$$

$$\sum_{\mu=1}^{p} \frac{\left(\sum_{k=1}^{m} n_k^* R_{k\mu}\right)^2}{s_\mu} \left(\sum_{i=1}^{m} v_i R_{i\mu} \sqrt{n_i^*}\right)^2 \geq 0,$$

so $D^{1/2} M D^{1/2}$ is symmetric and positive semi-definite, from which it follows that $D^{1/2} M D^{1/2}$ and thus DM has nonnegative eigenvalues.

Using Lemma 4, we can determine whether all species coexist in a community of p resources and m species with arbitrary enzyme budgets $E_i = \sum_{\mu=1}^{p} R_{i\mu}$ and equal death rates d. However, in the case that not all species in the community $\mathcal{C}$ coexist, we can determine which subset $\mathcal{S} \subseteq \mathcal{C}$ of species coexists as follows.

A subset of species $\mathcal{S} \subseteq \mathcal{C}$ will coexist if there exist steady-state nutrient concentrations $c_\mu^* > 0$ such that $\sum_{\mu=1}^{p} R_{i\mu} c_\mu^* = d$ and $\hat{s}$ lies in the convex hull of the vectors $\vec{R}_i$, and no other species $j \in \mathcal{C} - \mathcal{S}$ can invade the coexisting subset $\mathcal{S}$.

We consider whether species $j \in \mathcal{C} - \mathcal{S}$ can invade the subset $\mathcal{S}$ by adding species j to the community of species in $\mathcal{S}$ at an infintesimal abundance $n_j \ll n_s$ for all $s \in \mathcal{S}$, and calculate dn_j/dt, the rate of change of abundance of species j. If the ratio $\frac{dn_j/dt}{n_j}$ is negative and is independent of n_j, then species j goes extinct, and therefore cannot invade the community and coexist. If the ratio $\frac{dn_j/dt}{n_j}$ is positive, then species j can invade the community and coexist.

Lemma 5: *Consider a set $\mathcal{C}$ of species with equal death rates d that coexist with steady state resource concentrations $c^*_\mu > 0$ such that $\sum_{\mu=1}^{p} R_{i\mu} c^*_\mu = d$ for all species $i \in \mathcal{C}$. If a new community $\mathcal{C}' = \mathcal{C} \cup \{j\}$ does not satisfy the requirements for all species in $\mathcal{C}'$ to coexist, then species j can invade the set of species in $\mathcal{C}$ if $\sum_{\mu=1}^{p} R_{i\mu}\, c^*_\mu > d$, and species j cannot invade the set of species in $\mathcal{C}$ if $\sum_{\mu=1}^{p} R_{i\mu}\, c^*_\mu < d$.*

Proof: Consider a set $\mathcal{C}$ of m coexisting species with p resources, i.e., there exist $c^*_\mu > 0$ such that $\sum_{\mu=1}^{p} R_{i\mu} c^*_\mu = d$ for all species $i \in \mathcal{C}$. Next, consider adding another species j creating a new set $\mathcal{C}' = \mathcal{C} \cup \{j\}$, where the set of species $\mathcal{C}'$ does not satisfy the requirements for all species in $\mathcal{C}'$ to coexist. Therefore, we know that at least one species in $\mathcal{C}'$ must go extinct. To determine if species $j \in \mathcal{C}'$ goes extinct, we calculate dn_j/dt when n_j is infinitesimal such that for all other species $i \in \mathcal{C}$, n_i are the steady state abundances just before species j was introduced. We first calculate the perturbed resource concentration c_μ to first order in $\epsilon_\mu \equiv n_j R_{j\mu} c^*_\mu / s_\mu \ll 1$,

$$c_\mu = \left(\frac{n_j R_{j\mu}}{s_\mu} + \frac{\Sigma_{k\in\mathcal{C}} n_k R_{k\mu}}{s_\mu}\right)^{-1} = \left(\frac{\epsilon_\mu}{c^*_\mu} + \frac{1}{c^*_\mu}\right)^{-1} \approx c^*_\mu (1 - \epsilon_\mu), \quad (14)$$

where c^*_μ is the concentration of resource μ when community $\mathcal{C}$ coexists at steady state, so that we can use Eq. 10. Plugging in Eq. 14 to calculate the approximate growth rate of species i,

$$g_i(\vec{n}) \approx \sum_{\mu=1}^{p} R_{i\mu}\, c^*_\mu (1 - \epsilon_\mu),$$

which is valid for $i \in \mathcal{C}$ or $i = j$. To determine whether species j can invade the community $\mathcal{C}$, we first use Eq. 1 and the above approximation to calculate the (approximate) ratio $\frac{dn_i/dt}{n_i}$ for $i \in \mathcal{C}$:

$$\frac{dn_i/dt}{n_i} \approx \sum_{\mu=1}^{p} R_{i\mu}\, c_\mu^*(1-\epsilon_\mu) - d = -\sum_{\mu=1}^{p}(R_{i\mu}c_\mu^*)\epsilon_\mu,$$

since $\sum_{\mu=1}^{p} R_{i\mu}c_\mu^* = d$. Next, we similarly calculate the same (approximate) ratio for species j,

$$\frac{dn_j/dt}{n_j} \approx \sum_{\mu=1}^{p} R_{i\mu}\, c_\mu^*(1-\epsilon_\mu) - d \approx \sum_{\mu=1}^{p} R_{i\mu}c_\mu^* - d, \qquad (15)$$

since $\sum_{\mu=1}^{p} R_{i\mu}c_\mu^* \neq d$. We analyze Eq. 15 separately for the two cases $\sum_{\mu=1}^{p} R_{j\mu}\, c_\mu^* < d$ and $\sum_{\mu=1}^{p} R_{j\mu}\, c_\mu^* > d$.

Case $\sum_{\mu=1}^{p} R_{j\mu}\, c_\mu^* < d$: Here, the ratio $\frac{dn_j/dt}{n_j}$ is negative and independent of n_j (i.e., order zero in ϵ_μ), and the ratio $\frac{dn_i/dt}{n_i}$ for $i \in \mathcal{C}$ is order $\epsilon_\mu \ll 1$. Therefore, species j cannot invade community $\mathcal{C}$, i.e. species j goes extinct, leaving all species in $\mathcal{C}$ able to coexist.

Case $\sum_{\mu=1}^{p} R_{j\mu}\, c_\mu^* > d$: Here, the ratio $\frac{dn_j/dt}{n_j}$ is positive and order zero in ϵ_μ, and the ratio $\frac{dn_i/dt}{n_i}$ for $i \in \mathcal{C}$ is negative and order $\epsilon_\mu \ll 1$. Therefore, species j can invade community $\mathcal{C}$, meaning that at least one species in $\mathcal{C}$ must go extinct, since $\mathcal{C} \cup \{j\}$ does not satisfy the condition for coexistence by assumption.

By combining Lemmas 3-5, we obtain the coexistence rules presented in the main text.

Closed-form solution for steady-state abundances of m coexisting species when competing for $p = m$ resources

When the m species satisfy the convex hull coexistence condition, we can solve for the unique steady state abundances $\vec{n}^*$. Starting from Eq. 6, we set the growth rate g_i equal to the death term $d + d_i$ for each species and write the equations in matrix form,

$$\overrightarrow{n^*}RC = \vec{s} \qquad (16)$$

$$R\vec{c}^T = \vec{d}^T \qquad (17)$$

where $R = \{R_{i\mu}\}$, $\vec{d} = (d + d_1, \dots, d + d_m)$, $C = \text{diag}(c_\mu^*)$, and $\vec{c} = (c_1^*, \dots, c_p^*)$. R is a square matrix since $m = p$, and all species have linearly independent consumption strategies so the rows of R are linearly independent, therefore R is invertible. Since the steady-state nutrient concentrations are positive and all resources are supplied at positive rates, the diagonal matrix C is positive along the diagonal, thus C is invertible. Therefore, we rearrange Eq. 16 and 17 as follows:

$$\vec{n}^* = \vec{s}C^{-1}R^{-1} \qquad (18)$$

where the diagonal elements of C are given by

$$\vec{c} = R^{-1}\vec{d}^T. \qquad (19)$$

Comparison of steady-state abundances for bactericidal versus bacteriostatic antibiotics when $m = p = 2$

By comparing the population dynamics with species-specific death rates d_i in Eq. 9 and species-specific resource consumption rate reduction factors b_i in Eq. 5, the steady-state abundances are not in general the same in these two scenarios despite the set of coexisting species being identical when $b_i = (d + d_i)/d$.

When $m = p = 2$, we can solve explicitly for the steady state abundances $\vec{n}^*$ using Eq. 18 and 19. We focus on a resource consumption rate matrix $R = ((1, \theta), (\theta, 1))$, dilution rate $d = 1$, and equal supply rates for both resources $\vec{s} = (0.5, 0.5)$.

Bactericidal antibiotics

We consider varying the death rate of species 1 d_1 from 0 to d_1^{max} while keeping the death rate of species 2 at zero. The death rate vector is therefore $\vec{d} = (d_1 + 1, 1)$ since the dilution rate d is set to 1. Plugging into Eq. 18 and 19, the steady state abundances are

$$\vec{n}_{\text{cidal}}^* = \frac{1}{2}\left(\frac{1}{d_1 + 1 - \theta} + \frac{\theta}{\theta + d_1\theta - 1}, \frac{1}{1 - d_1\theta - \theta} + \frac{\theta}{\theta - d_1 - 1}\right). \qquad (20)$$

We can solve for the maximum death rate of species 1 d_1^{max}, the death rate above which species 1 goes extinct, by solving for the death rate such that the steady-state abundance of species 1 $n_{1,\text{cidal}}^*$ is zero:

$$d_1^{\max} = \frac{\theta^2 - 2\theta + 1}{2\theta}.$$

Bacteriostatic antibiotics

We next consider reducing the consumption rates of species 1 by a factor b_1, giving the new consumption rate matrix, $R = \big((1/b_1, \theta/b_1), (\theta, 1)\big)$, with zero death aside from the dilution of the chemostat so $\vec{d} = (1,1)$. To directly compare the bacteriostatic and bactericidal cases, we take $b_1 = (d + d_1)/d$, motivated by the fact that the coexistence is identical in bacteriostatic and bactericidal cases with this relationship between b_1 and d_1. Plugging into Eq. 18 and 19, the steady state abundances are

$$\vec{n}^*_{static} = \frac{1}{2}\left(\frac{d_1+1}{d_1+1-\theta} + \frac{\theta d_1 + \theta}{\theta + d_1\theta - 1}, \frac{1}{1-d_1\theta-\theta} + \frac{\theta}{\theta - d_1 - 1}\right). \quad (21)$$

By comparing Eq. 20 and 21, the steady state abundance of the non-targeted species 2 is the same in the bactericidal and bacteriostatic cases (i.e. $n^*_{2,\text{cidal}} = n^*_{2,\text{static}}$, Fig. S1A). However, for species 1, in the range $d_1 \in [0, d_1^{\max}]$,

$$n^*_{1,\text{static}} = \frac{1}{2}\left(\frac{d_1+1}{d_1+1-\theta} + \frac{\theta d_1 + \theta}{\theta + d_1\theta - 1}\right) = n^*_{1,\text{cidal}} + \frac{1}{2}\left(\frac{d_1}{d_1+1-\theta} + \frac{\theta d_1}{\theta + d_1\theta - 1}\right) \geq n^*_{1,\text{cidal}},$$

where equality occurs when $d_1 = 0$ or $d_1^{\max}$. In other words, the community is more even at steady state during treatment with bacteriostatic antibiotics (Fig. S1A). Notably, the difference in steady-state abundance of the targeted species during bactericidal versus bacteriostatic antibiotics decreased when θ increased; larger θ corresponds to a larger degree of resource competition. This finding is consistent with the behavior at the extreme limits: when there is no resource competition, the steady-state abundances are unaffected by reducing the resource consumption rates for one species, but varying the death rate of one species must change the steady-state abundances regardless of the degree of resource competition.

Condition for maximizing the effective number of species at steady state N_{eff}

Consider m species competing for $p = m$ resources. To maximize N_{eff}, we solve for the strategies $R_{i\mu}$ such that $dn_i/dt = 0$ at steady-state abundances $n_i^* = 1/N$, where $N = \sum_{i=i}^{p} n_i^*$. Plugging these conditions into Eq. 6 and defining the fractional consumption rate $F_{i\mu} \equiv R_{i\mu} / \sum_{k=1}^{m} R_{k\mu}$,

$$d + d_i = \sum_{\mu=1}^{p} \frac{R_{i\mu} s_\mu}{\Sigma_k n_k R_{k\mu}} = \frac{1}{N} \sum_{\mu=1}^{p} \frac{R_{i\mu} s_\mu}{\Sigma_k R_{k\mu}} = \frac{1}{N} \sum_{\mu=1}^{p} F_{i\mu} s_\mu .$$

When all resources are supplied at equal rates $s_\mu = 1/S$, the condition for maximizing N_{eff} becomes $\sum_{j=\mu}^{p} F_{i\mu} = \delta_i$ where $\delta_i = (d + d_i)NS$ is a constant. When all species have uniform death rate, the condition for maximizing N_{eff} becomes $\sum_{\mu=1}^{p} F_{i\mu} = F_i = F$, which can be interpreted simply as the total fractional consumption rate F_i being constant for all species.

Application of the coexistence rules to calculate steady-state abundances when $\boldsymbol{m = p = 3}$

When $m = p = 3$, steady-state abundances can be calculated without simulating population dynamics and instead by applying coexistence rules. From Eq. 6, the steady-state abundances n_i^* satisfy $S = n_1^*(d + d_1) + \cdots + n_m^*(d + d_m)$, from which it follows that if species 1 is the only existing species at steady state, then $n_1^* = S/(d + d_1)$, and if species 1 and 2 are the only coexisting species at steady state, then $n_2^* = \frac{S - n_1^*(d+d_1)}{d+d_2}$. Thus, we can simplify the calculation of steady-state abundances and avoid calculating the full trajectory of population dynamics by using the following algorithm to calculate steady-state abundances.

1. Check if all species coexist: We solve for the steady-state abundances using Eq. 18 and 19, and if all steady state abundances are positive then all species can coexist, satisfying the assumptions of Eq. 18 and 19. If Eq. 18 and 19 yield steady-state abundances that are not all positive, then at least one species must go extinct and therefore Eq. 18 and 19 are not valid.

2. Check if exactly one species exists: Species i is the sole existing species if the other two species cannot invade species i at steady state. Thus, we calculate the steady-state nutrient concentrations such that species i is the sole existing species using Eq. 10, giving $c_\mu^* = s_\mu(d + d_i)/(SR_{i\mu})$, using the fact that $n_i^* = S/(d + d_i)$ in this case. Next, we use Eq. 3 to calculate g_j, the growth rate of species $j \neq i$, and if $g_j < d_j$ then j cannot

invade i; otherwise, j can invade i. If there is a species i such that all other species $j \neq i$ cannot invade i, then species i is the only existing species at steady state and its abundance is $n_i^* = S/(d + d_i)$.

3. Check if exactly two species coexist: After ruling out that three or one species coexist, we know that two species must coexist. The pair of coexisting species must be mutually invasible, meaning for example that species 1 can invade species 2 and vice versa. We say that species 1 can invade species 2 if, when species 2 is in monoculture at steady state and then perturbed by the addition of species 1 at infinitesimal abundance, the growth rate of species 1 is positive. We check this mutual invasibility condition for all three pairs of species. For a pair of species that is mutually invasible (here taken to be species 1 and 2), we assume these are the two species that coexist and calculate their steady-state abundances using the relation $n_2^* = \frac{S - n_1^*(d+d_1)}{d+d_2}$ and Eq. 6 to solve for n_1^* such that dn_1/dt is zero. The outcome is an order 3 polynomial in n_1^* with two roots of n_1^* equal to zero or $S/(d + d_1)$. We solve for the third root (which must be between zero and $S/(d + d_1)$). After doing so, we use Eq. 3 to calculate the resource concentrations at steady state (assuming that the pair of species are the two that coexist), and test whether the third species cannot invade. If the third species cannot invade, then we have determined the two coexisting species and calculated their steady-state abundances. If the third species can invade, then these two are not the pair of coexisting species.

Generalizing to more diverse communities

Consider m species and p resources, with symmetry among $x \leq m$ species such that for two species $k, k' \in \{1, \ldots, x\}$, the abundance of species k equals the abundance of species k': $n_k(t) = n_{k'}(t)$, for all time t. We then replace all x species $k \in \{1, \ldots, x\}$ with a single "aggregate" species k^*, such that the rate at which species k^* consumes resource μ is $R_{k^*\mu} \equiv \frac{1}{x}\Sigma_{k=1}^{x} R_{k\mu}$ and the death rate $d_{k^*} \equiv d_1$, where by symmetry $d_1 = d_2 = \cdots = d_x$.

We first prove by induction that if the initial abundance of the aggregate species equals the sum of the initial abundance of species $1, \ldots, x$, then the abundance of the aggregate

species equals the sum of the abundance of species $1, \dots, x$ for all time. That is, if $n_{k^*}(t=0) = \Sigma_{k=1}^{x} n_k(t=0)$, then $n_{k^*}(t) = \Sigma_{k=1}^{x} n_k(t)$ for all time t. We assume that at some time t', $n_{k^*}(t') = \Sigma_{k=1}^{x} n_k(t')$, and show that $dn_{k^*}/dt = \Sigma_{k=1}^{x} dn_k/dt$ at time t. From Eq. 1,

$$\frac{dn_{k^*}}{dt} = n_{k^*}(t)\big(g_{k^*} - (d + d_{k^*})\big),$$

where

$$g_{k^*} = \sum_{\mu=1}^{p} R_{k^*\mu} c_\mu = \sum_{\mu=1}^{p} \left(\frac{1}{x}\sum_{k=1}^{x} R_{k\mu}\right) c_\mu = \frac{1}{x}\sum_{k=1}^{x}\sum_{\mu=1}^{p} R_{k\mu} c_\mu = \frac{1}{x}\sum_{k=1}^{x} g_k = g_{k'}$$

for any $k' \in \{1, \dots, x\}$ by symmetry among species $k \in \{1, \dots, x\}$. Thus, along with the relation $d_{k^*} = d_k$ for any $k \in \{1, \dots, x\}$, we have $dn_{k^*}/dt = \sum_{k=1}^{x} n_k(t)\big(g_k - (d + d_k)\big) = \sum_{k=1}^{x} dn_k\,/dt$ at time t, from which we conclude that if $n_{k^*}(t=0) = \Sigma_{k=1}^{x} n_k(t=0)$, then $n_{k^*}(t) = \Sigma_{k=1}^{x} n_k(t)$ for all time t.

Next, the concentration of resource μ is

$$c_\mu = s_\mu \left(\sum_{k=1}^{x} n_k R_{k\mu}\right)^{-1} = s_\mu \left(\sum_{k=1}^{x} n_k R_{k\mu} + \sum_{k=x+1}^{m} n_k R_{k\mu}\right)^{-1}$$

where

$$\textstyle\sum_{k=1}^{x} n_k R_{k\mu} = \sum_{k=1}^{x} \left(\frac{1}{x}\sum_{k'=1}^{x} n_{k'}\right) R_{k\mu} = \left(\sum_{k=1}^{x} n_{k'}\right)\left(\frac{1}{x}\sum_{k=1}^{x} R_{k\mu}\right) = n_{k^*} R_{k^*\mu}.$$

Therefore, the concentration of each resource μ is unchanged if all species $k \in \{1, \dots x\}$ are replaced by a single aggregate species k^*. Hence, the abundance of each species $j \in \{x+1, \dots, m\}$ is also unchanged by this replacement, since Eq. 1 for species j is unaffected.

Similarly, consider m species and p resources, with symmetry among $y \le p$ resources such that for two resources $\mu, \mu' \in \{1, \dots, y\}$, $c_\mu(t) = c_{\mu'}(t)$ for all time t and for each species k, $R_{k\mu} = R_{k\mu'}$. We replace all resources $\mu \in \{1, \dots, y\}$ with a single "aggregate" resource μ^*, where the supply rate of resource μ^* is $s_{\mu^*} \equiv \sum_{\mu=1}^{y} s_\mu = y s_1$, by symmetry among resources $\mu \in \{1, \dots, y\}$, and the rate at which species k consumes resource μ^* is $R_{k\mu^*} \equiv R_{k\mu}$ for arbitrary $\mu \in \{1, \dots, y\}$. Under this replacement, Eq. 3 dictates that $c_{\mu^*} =$

$s_{\mu^*}\left(\sum_{k=1}^{m} n_k R_{k\mu^*}\right)^{-1} = \sum_{\mu=1}^{y} s_\mu \left(\sum_{k=1}^{m} n_k R_{k\mu}\right)^{-1}$ based on the definitions of s_{μ^*} and $R_{k\mu^*}$. Therefore, $c_{\mu^*} = \sum_{\mu=1}^{y} c_\mu$. Moreover, Eq. 2 dictates that $g_k = \sum_{\mu=1}^{p} R_{k\mu}\, c_\mu = \sum_{\mu'=1}^{y} R_{k\mu'}\, c_{\mu'} + \sum_{\mu=y+1}^{p} R_{k\mu}\, c_\mu$, where $\sum_{\mu'=1}^{y} R_{k\mu'}\, c_{\mu'} = R_{k\mu^*} \sum_{\mu'=1}^{y} c_{\mu'} = R_{k\mu^*} c_{\mu^*}$. Therefore, the growth rate g_k is unchanged, and thus all species abundances are unchanged.

The above analysis shows that multiple non-targeted species with distinct but symmetrical niches can be grouped into effectively a single non-targeted species with a niche of effectively one resource. Similarly, in the case that a broad-spectrum antibiotic is used to target multiple species with symmetrical consumption niches, the targeted species can be represented as an effective single species being targeted by a narrow-spectrum antibiotic. In this sense, our analyses in the main text generalize to larger community structures obeying this symmetry.

Extending the model to incorporate metabolic cross-feeding

To incorporate cross-feeding into the CR model, we assumed that when species k consumes resource μ, only the fraction $(1 - L_\mu)$ of the mass of resource μ contributes to growth of species k and the remaining fraction L_μ is excreted by the cells as metabolic byproducts, as in Ref. 31. For simplicity, we assumed that these metabolic byproducts represent a uniform distribution of all supplied resources. With these p additional parameters, $0 \le L_\mu < 1$, the population dynamics described by Eq. 2 become $g_i = \sum_{\mu=1}^{p} (1 - L_\mu) R_{i\mu} c_\mu$, modifying Eq. 3 governing the resource concentrations to

$$dc_\mu/dt = s_\mu - dc_\mu - \sum_{i=1}^{m} n_i R_{i\mu} c_\mu + \sum_{j=1}^{m} \sum_{\mu'=1}^{p} n_j R_{j\mu'}\, c_{\mu'} L_{\mu'} (1/p).$$

Removing the dilution term as before and setting $dc_\mu/dt = 0$, we obtain

$$c_\mu = \frac{s_\mu + \xi}{\sum_{i=1}^{m} n_i R_{i\mu}},$$

where $\xi \equiv \frac{1}{p} \sum_{j=1}^{m} n_j \sum_{\mu'=1}^{p} L_{\mu'} R_{j\mu'}\, c_{\mu'}$. This relation modifies Eq. 6 to include cross-feeding as

$$dn_i/dt = n_i \left(\sum_{\mu=1}^{p} \frac{R_{i\mu}[(1 - L_\mu)(s_\mu + \xi)]}{\sum_{j=1}^{m} n_j R_{j\mu}} - (d + d_i) \right).$$

The equation above is identical to Eq. 6 under the transformation of resource supply rate vector $s_\mu \to s'_\mu \equiv (1 - L_\mu)(s_\mu + \xi)$. Therefore, if all resources are supplied at equal rates ($s_\mu = S/p$), then ξ simply scales the steady-state species abundances and does not change which species coexist, since ξ is independent of μ. Hence, we can set ξ to zero without affecting coexistence and calculate the effective supplied resource rates $s'_\mu = (1 - L_\mu)s_\mu$ to solve for the steady-state species abundances with these effective supplied resource rates.

SUPPLEMENTARY FIGURES

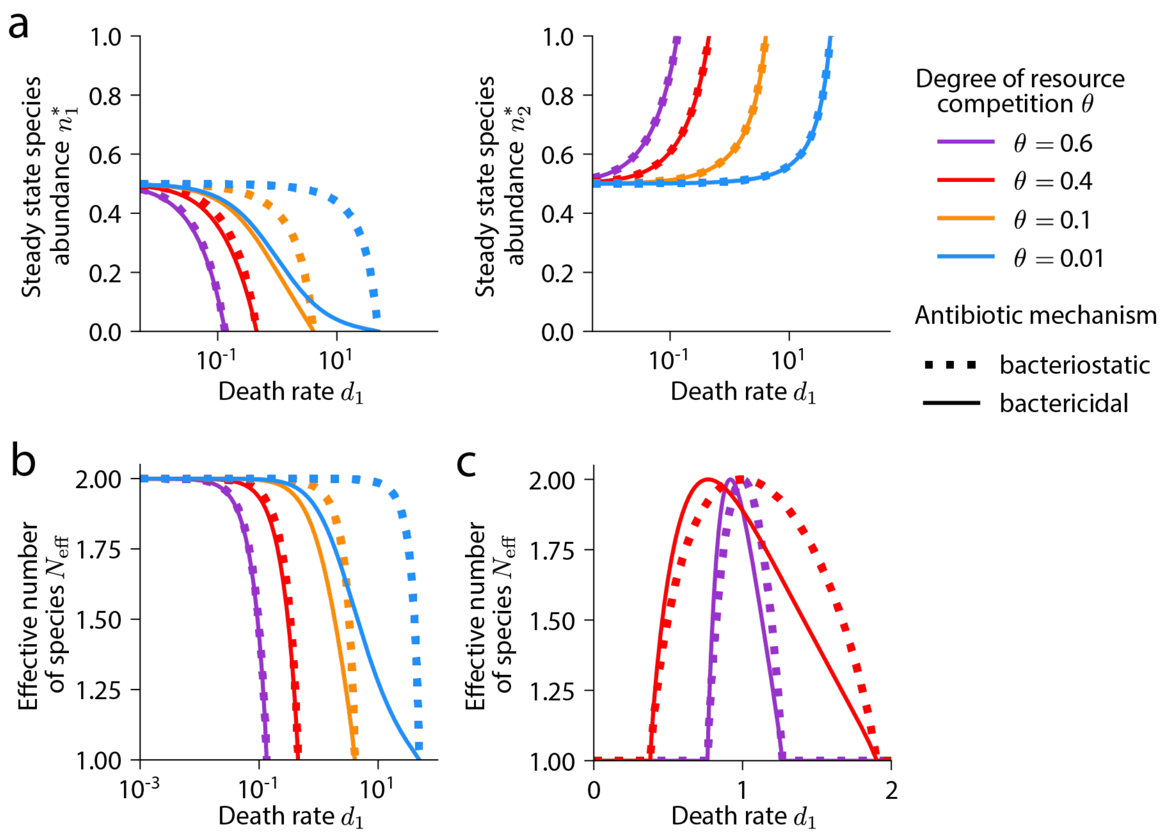

Figure S1: Bactericidal and bacteriostatic antibiotics have similar effects on species abundances.

a) Simulations of a community with $m = p = 2$ defined by the resource consumption rate matrix $R = ((1, \theta), (\theta, 1))$, where θ parametrizes the niche overlap between the two species. Species 1 was targeted with increasing death rate d_1. (Left) The steady state abundance of species 1 (n_1^*) during treatment with a bactericidal antibiotic was always lower in comparison with the corresponding perturbation with a bacteriostatic antibiotic, given by $b_1 = (d + d_1)/d$ (Supplemental Text). (Right) The steady-state abundance of species 2 (n_2^*) was independent of the antibiotic mechanism.

b) For the community in (a), N_{eff} was generally higher at steady state for bacteriostatic versus bactericidal antibiotics.

c) For a community defined by the resource consumption rate matrix $R = ((2, 2\theta), (\theta, 1))$, steady-state evenness N_{eff} was non-monotonic (Supplemental

Text), analogous to the richness in Fig. 2a. The difference in N_{eff} between a bacteriostatic and bactericidal antibiotic was greater when there was less niche overlap ($\theta = 0.4$).

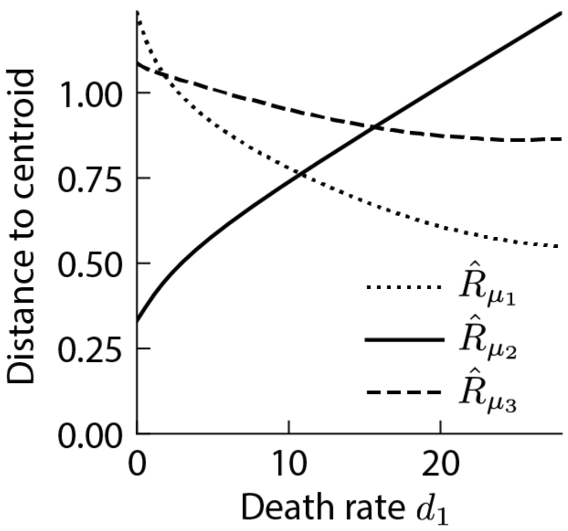

Figure S2: The centroid of the coexistence region shifts towards the niche of the targeted species during antibiotic treatment.

For the community in Fig. 3a, we plotted the distance between the centroid of the coexistence region and each corner of the simplex, corresponding to each consumption niche. The niche of the targeted species (blue) is resources 1 and 3, so targeting the blue species caused the non-targeted species to encroach on the niche of the targeted species, as indicated by the centroid of the coexistence region moving closer to the corners of the simplex corresponding to resources 1 and 3, and farther from resource 2.

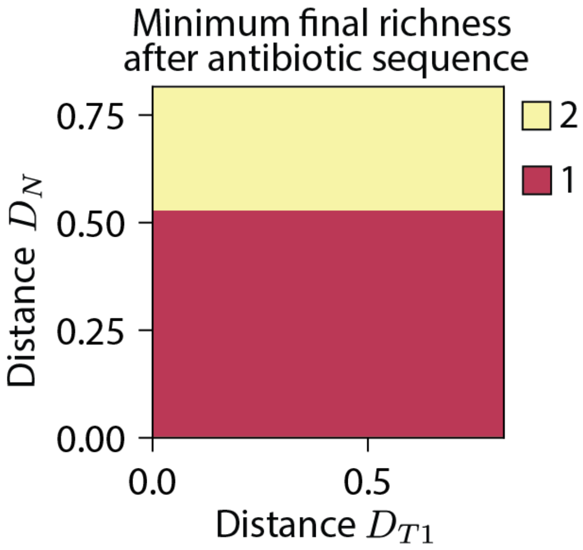

Figure S3: Final richness after antibiotic sequencing is always ≥ 2 when $D_N \gtrsim 0.5$, precluding non-transitivity.

We calculated the final richness after all antibiotic sequences (and reverse sequences) across the parameters (D_{T1}, D_{T2}, D_N, d_1, d_2). The minimum final richness after all antibiotic sequences with $D_N \gtrsim 0.5$ was 2. For $\Delta\rho$ to be nonzero (non-transitivity), the final richness after an antibiotic sequence must be 1, indicating that non-transitivity is not possible when $D_N \gtrsim 0.5$.

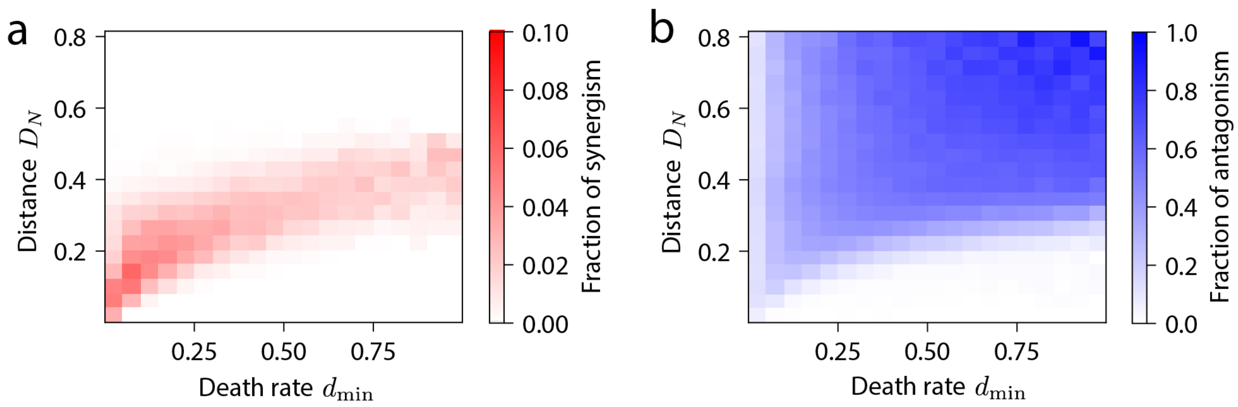

Figure S4: Non-additivity requires a balance between D_N and death rates.

a) For communities with higher D_N, representing a more specialized non-targeted species, the fraction exhibiting synergism was maximal at larger values of $d_{\min}$ (the smaller of the two death rates).

b) Communities with higher $d_{\min}$, the fraction exhibiting antagonism increased with increasing D_N.

These simulations generalize the results in Fig. 5h in the main text to communities without symmetry in consumption rates.

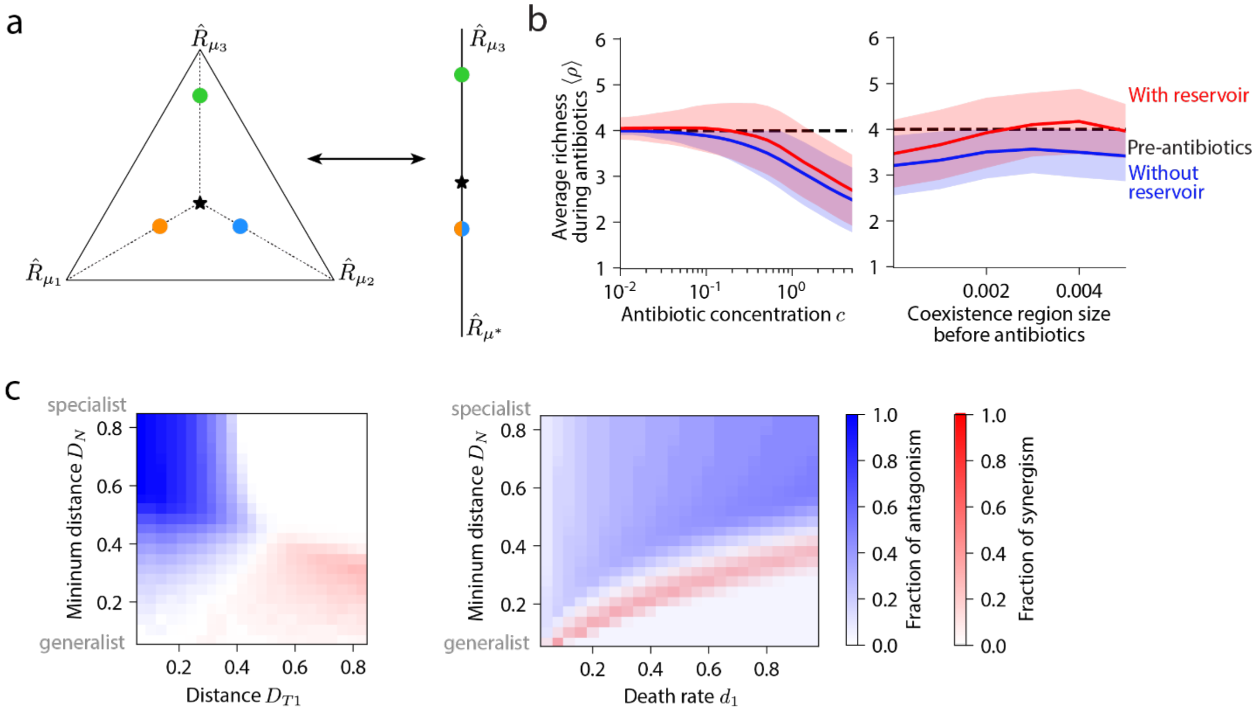

Figure S5: Non-additivity occurs in large communities.

a) The dynamics of a community with multiple non-targeted species with symmetric consumption niches (blue and orange, left) can be reduced to those of a community with one effective non-targeted species (right) (Supplemental Text).

b) Larger communities exhibit similar responses to antibiotic perturbation as three-species communities. Numerical simulations analogous to those in Fig. 2d were carried out for communities that are twice as diverse, with 6 resources and 6 species, 4 of which coexist before antibiotic perturbation. Shading denotes one standard deviation from the mean with a cutoff at a maximum richness of 4 for simulations without a re-seeding reservoir.

c) Non-additivity occurs in larger communities under similar parameter regimes as for three-species communities. Numerical simulations analogous to those in Fig. 5g,h were carried out for communities with 4 resources and 4 species, 2 of which are non-targeted. The minimum distance D_N was defined as the smaller of the two distances from each of the non-targeted species to the supplied resource point.

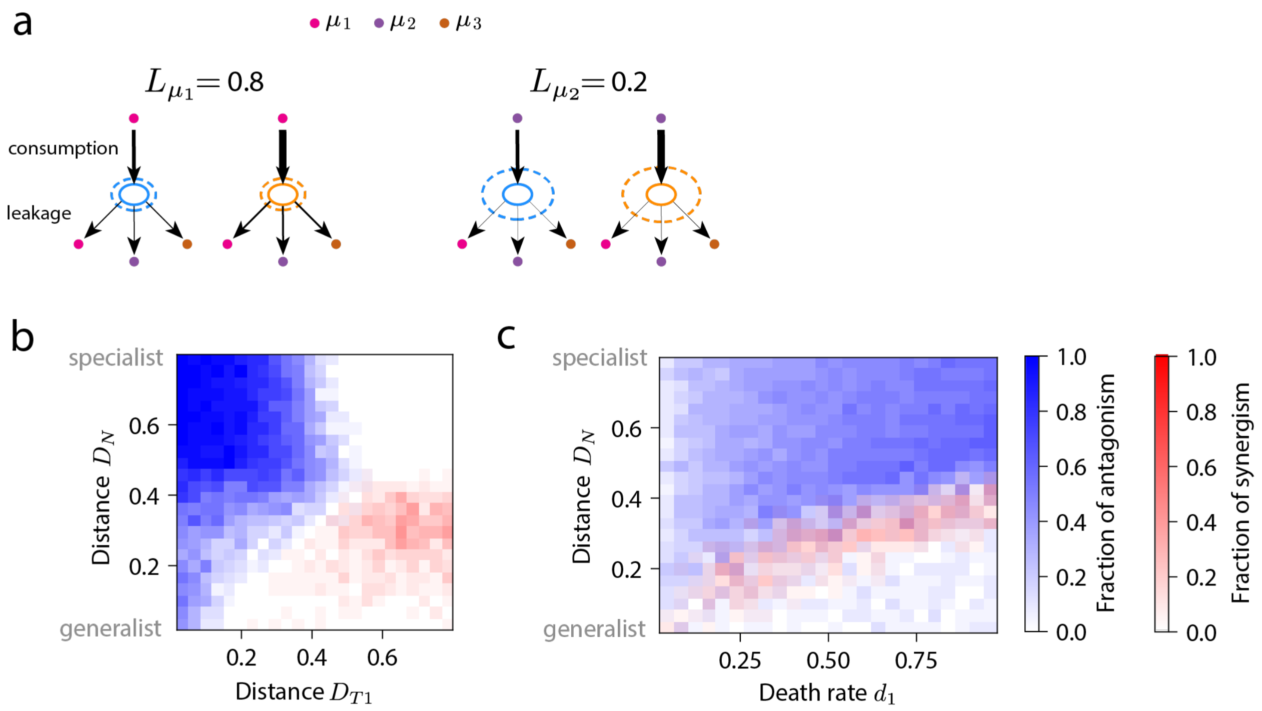

Figure S6: Non-additivity occurs in communities with cross-feeding.

a) Implementation of cross-feeding via uniform leakage[31] (Supplemental Text). The leakage fraction L_μ of resource μ describes the fraction of flux that is secreted as other resources (arrows) instead of contributing to biomass growth (dashed circles). We assumed that the leaked fraction contributes uniformly to all resources, and that the leakage fraction for each resource is the same across species.

b) Non-additivity occurs in communities with cross-feeding under similar parameter regimes as for three-species communities. Numerical simulations analogous to those in Fig. 5g,h were carried out for three-species communities with a moderate level of uniform cross-feeding, in which leakage fractions L_μ were drawn uniformly between 0 and 0.5.
